# Intracellular and Intercellular Signalling Mechanisms following DNA Damage Are Modulated By PINK1

**DOI:** 10.1155/2018/1391387

**Published:** 2018-06-27

**Authors:** Mihaela Temelie, Diana Iulia Savu, Nicoleta Moisoi

**Affiliations:** ^1^Department of Life and Environmental Physics, Horia Hulubei National Institute of Physics and Nuclear Engineering, Reactorului 30, P.O. Box MG-6, 077125 Magurele, Romania; ^2^Leicester School of Pharmacy, Faculty of Health Sciences, De Montfort University, The Gateway, Hawthorn Building 1.03, Leicester LE1 9BH, UK

## Abstract

Impaired mitochondrial function and accumulation of DNA damage have been recognized as hallmarks of age-related diseases. Mitochondrial dysfunction initiates protective signalling mechanisms coordinated at nuclear level particularly by modulating transcription of stress signalling factors. In turn, cellular response to DNA lesions comprises a series of interconnected complex protective pathways, which require the energetic and metabolic support of the mitochondria. These are involved in intracellular as well as in extracellular signalling of damage. Here, we have initiated a study that addresses how mitochondria-nucleus communication may occur in conditions of combined mitochondrial dysfunction and genotoxic stress and what are the consequences of this interaction on the cell system. In this work, we used cells deficient for PINK1, a mitochondrial kinase involved in mitochondrial quality control whose loss of function leads to the accumulation of dysfunctional mitochondria, challenged with inducers of DNA damage, namely, ionizing radiation and the radiomimetic bleomycin. Combined stress at the level of mitochondria and the nucleus impairs both mitochondrial and nuclear functions. Our findings revealed exacerbated sensibility to genotoxic stress in PINK1-deficient cells. The same cells showed an impaired induction of bystander phenomena following stress insults. However, these cells responded adaptively when a challenge dose was applied subsequently to a low-dose treatment to the cells. The data demonstrates that PINK1 modulates intracellular and intercellular signalling pathways, particularly adaptive responses and transmission of bystander signalling, two facets of the cell-protective mechanisms against detrimental agents.

## 1. Introduction

Mitochondria have crucial functions in the cell, including ATP generation, iron-sulfur cluster biogenesis, nucleotide biosynthesis, metabolism, calcium homeostasis, and cell death regulation. In neurons, mitochondria are critical for the maintenance of membrane ion gradients, neurotransmission, and synaptic plasticity. Most neuronal ATP is generated through oxidative phosphorylation in the mitochondria, only about 10% being produced by glycolysis. Neuronal functions require tight regulation on mitochondrial activity and homeostasis. As mitochondrial biogenesis is controlled by the nucleus and most mitochondrial proteins are encoded by nuclear genes, a tight communication network between mitochondria and the nucleus has evolved, comprising signalling cascades, proteins with dual localization to the two compartments, and sensing of mitochondrial products by nuclear proteins [1]. These enable an organellar crosstalk that facilitates integration of cellular and environmental signals to adjust their function for the maintenance of cellular homeostasis particularly at functional checkpoints in the cellular life.

Mitochondrial dysfunction refers broadly to an extended plethora of defects covering accumulation of unfolded proteins in the mitochondrial matrix and conditions that impair OXPHOS activity such as genotoxic stress and chemical stressors. Mitochondria cope with endogenous and exogenous stress factors through molecular and organellar mitochondrial quality control (mtQC) in the form of mitochondrial unfolded protein response (mtUPR), fusion and fission processes, and mitophagy. The mtUPR is a transcriptional response that is initiated by mitochondrial dysfunction and involves mitochondria-nucleus communication and is regulated at multiple levels including transcription and chromatin remodeling [2, 3]. Organellar mitochondrial quality control, also known as mitophagy, has been extensively studied in the last ten years. The current prevailing model proposes that PINK1 and Parkin constitute the main system for sensing and modulating removal of dysfunctional mitochondria. Thus, under “basal conditions” in healthy mitochondria, the phosphatase and tensin homolog- (PTEN-) induced kinase 1 kinase (PINK1) is fully imported into the mitochondrial matrix where it is rapidly degraded by proteolysis [4, 5]. In conditions of mitochondrial stress and loss of mitochondrial membrane potential, PINK1 accumulates in full form in the outer mitochondrial membrane (OMM), with its kinase domain facing the cytoplasm. In this form, it is involved in the process of mitophagy that implicates a series of reciprocal biochemical processes involving the E3-ligase PARKIN. Thus, PINK1 phosphorylates PARKIN on the OMM, and in turn Parkin ubiquitinates PINK1 and other mitochondrial proteins present on the OMM preparing the damaged mitochondria for undergoing lysosomal degradation [6–8].

Loss-of-function mutations of PINK1 and Parkin lead to the early onset of Parkinson's disease (PD) [9]. Various cellular parameters and biochemical pathways have been analyzed with respect to the PINK1 role in mitochondrial homeostasis. Besides its well-documented role in mitophagy, the kinase is involved in several other mitochondrial-related pathways including ATP production, calcium homeostasis, and apoptosis [10]. Recent work has uncovered additional functions of PINK1 besides the maintenance of mitochondrial function. Thus, impairment of mitochondrial function determined by PINK1 or Parkin loss of function is signaled to the endoplasmic reticulum (ER), the mitochondria-ER signalling enhancing neurodegeneration [11]. Moreover, PINK1 is also involved in aging [12] and could have oncogenic potential (through the protection of healthy mitochondria in cancer cells) [13]. Therefore, deciphering the plethora of PINK1 functions would progress the understanding of the role of mitochondria in complex phenomena such as aging and age-related diseases.

Along with mitochondria, nuclear DNA integrity has a pivotal role in determining the fate of the cells challenged throughout the life with endogenous threats (ROS, DNA replication errors) as well as exogenous stress comprising physical and chemical agents. In response to stress insults that induce DNA lesions, mammalian cells activate a complex cell defense system, the DNA damage response (DDR) designed to detect the DNA lesions, and trigger signal transduction pathways that lead to either DNA repair, cell cycle delay, apoptosis, or senescence [14, 15]. Unrepaired DNA lesions may either result in cell death or can be a major source of genomic instability [15–17].

Numerous studies have shown that exposure of a biological system to low-level physical or chemical stress delivered as a single dose or cumulative repeated doses (priming treatment) induces changes that are able to prepare the biological system to respond to subsequent stressing factors (challenge treatment) in a way that leads to the accumulation of damage at a level that is lower than the level of damage induced by the challenge treatment alone (reviewed in [18]). This induced resistance to stress stimuli is a general defense mechanism called adaptive response (AR). Adaptive responses may be viewed as a special hormetic, benefic response. Hormesis is the first response after exposure to adaptive response doses, and some of the stimulatory effects then result in adaptive response to subsequent high-dose stress challenge. AR have been observed in a variety of endpoints including cell survival, gene mutations, chromosome aberrations and micronuclei induction, neoplastic transformation *in vitro*, and DNA single- and double-strand breaks.

Apart from these intracellular signals, the damaged cells send intercellular signals to their neighbour cells. The signals can be transmitted through gap junction for cells in direct contact or through the release of soluble factors in the extracellular media. The messenger molecules include ROS, nitric oxide, and cytokines as well as DNA and RNA molecules. These nontreated cells respond to signals produced by directly treated cells through a signalling process that has been termed the bystander effect (BE) [19–23]. The endpoints that demonstrate a BE address similar DNA damage and cell survival-cell death as in the case of AR. BE were first considered detrimental secondary effects of radiation exposure, given their manifestation as increased DNA damage, chromosomal aberration, and increased apoptosis in neighboring unexposed cells that may cumulate to induce either cellular death or tumorigenesis [22–26]. However, this view has been challenged by evidence that BE may represent a beneficial, adaptive phenomenon [26]. Mitochondria are associated with nontargeted effects induced by genotoxic agents, including adaptive response, hormesis, bystander effects, and genomic instability [27–29].

Mitochondrial dysfunction, the accumulation of genetic damages, and perturbed intercellular communication are recognized hallmarks of aging [30, 31] that are also closely associated with neurodegenerative disorder, cancer, and cardiovascular disease [32–35]. Analyzing the interaction of molecular cell changes with internal and external environment will allow the improvement of the understanding of the mechanisms underlying the hallmarks of aging.

Very few studies address the PINK1 contribution to mitochondria-nucleus communication. A recent phosphoproteomic study revealed that phosphorylation of 43% of nuclear proteins (transcription factors and proteins involved in DNA and RNA metabolism) is modulated by PINK1 [36]. Basal levels of PINK1 are low, due to its high turnover rate. Therefore, such a significant alteration in nuclear proteins determined by the kinase deficiency suggests a much wider role of the kinase. Thus, we believe that PINK1 involvement in mitochondria-nucleus signalling deserves higher consideration. Other areas that remain to be explored concern the role of PINK1 in intracellular and intercellular signalling following cellular DNA stress.

Here, we have undertaken a study whereby we use a combination of mitochondrial dysfunction comprising PINK1 loss of function and induction of DNA damage with the radiomimetic bleomycin or with X-ray irradiation. Using this model, we have addressed how mitochondria and the nucleus operate together to integrate cellular and oxidative stress signals for the maintenance of cellular homeostasis. In this study, we have identified key features of mitochondria-nucleus communications in intracellular and intercellular signalling emphasizing how their perturbation may lead to premature aging and neurodegenerative disorders.

## 2. Results

In order to address mitochondria-nucleus communication in the maintenance of cellular homeostasis during stress condition, we have employed a model of combined mitochondria and genotoxic stress. We have employed cellular models of mitochondrial dysfunction comprising PINK1 loss of function, namely, mouse embryonic fibroblasts (MEFs) derived from mice knockout (KO) for the *PINK1* gene [37] and the neuroblastoma dopaminergic cell line SH-SY5Y where the PINK1 expression was knocked down (KD) by RNA interference [38] together with their wild type (WT) and scrambled control (SC) lines. We have first confirmed the genotype of the cell lines and a consequent reduction of about 50% in basal ATP levels associated with the *PINK1* loss of function (Supplementary Figure S1). We have combined these known models of mitochondrial dysfunction with the induction of genotoxic stress using the chemical radiomimetic bleomycin (BLM) or X-ray irradiation treatment. The choice of the genotoxic treatments was made considering that throughout lifetime, the human body is exposed to DNA damage factors (ionizing radiation, chemotherapeutic agents) at low levels through environmental exposures (natural radiation on the earth surface, industrial radioactive materials) and at higher levels in the context of medical exposures for diagnostic and/or treatment [39].

This is the case for both types of genotoxic exposures that we have employed, chemical radiomimetic and X-ray irradiation. Thus, we have undertaken the studies at low/medium doses of treatment that are not inducing high levels of cell death. In addition, this adapted model enables us to reveal new mechanisms of nucleus and mitochondria communication that may allow cells to cope with environmental stress signals.

### 2.1. PINK1 Loss of Function Enhances Cellular Sensitivity to DNA Damage

**Figure 1:**
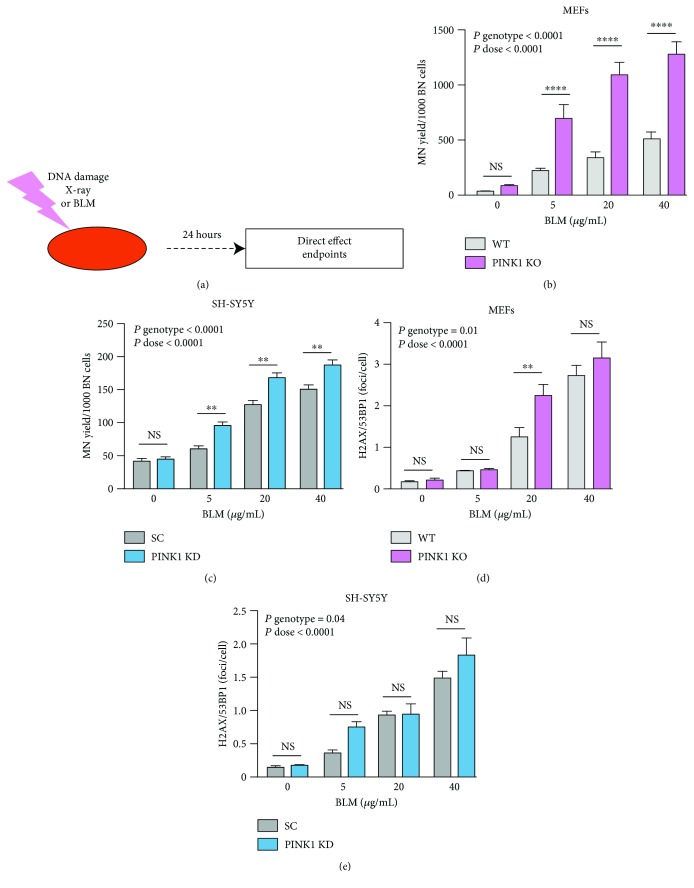
PINK1 loss of function enhances cellular sensitivity to DNA damage induced by BLM. (a) Schematic design of the study of DNA damage induced by genotoxic agents. The cells were exposed to genotoxic treatment (BLM for 1 h or X-rays) and incubated with fresh medium. At 24 h, cells were analyzed for the direct effects. Treatment of MEFs (b, d) and SH-SY5Y (c, e) with the DNA-damaging agent BLM at the indicated concentrations induces increased accumulation of MN (b, c) and *γ*H2AX/53BP1 foci (d, e) as DNA damage endpoint indicators. PINK1-KO MEFs and PINK1-KD SH-SY5Y cells present higher sensitivity in the accumulation of both types of DNA damages with MEF cells being more chemosensitive than SH-SY5Y. Each data point represents the mean ± SEM of at least three independent experiments. Statistical analysis is performed with two-way ANOVA with multiple comparisons, and *P* values are indicated in the figure. ^∗∗^ *P* < 0.01 and ^∗∗∗∗^ *P* < 0.0001.

First, we addressed how mitochondria contribute to the maintenance of DNA integrity under DNA damage conditions. The frequency of micronuclei (MN) induction and the occurrence of DNA double-strand breaks assessed as *γ*H2AX/53BP1 foci were the endpoints characterizing the extent of the genotoxic effect following the direct treatment with BLM and X-rays (Supplementary Figures S2 and S3 and Figure 1(a)).

Direct BLM treatment induced a dose-dependent increase in MN frequency in MEF cells. This increase occurred at a much higher level in PINK1-KO cells (Figure 1(b)). Similar results were obtained for neuroblastoma, but in this case, the differences between phenotypes are not so pronounced (Figure 1(c)). The neuronal SH-SY5Y cell line demonstrated higher resistance to the treatment as compared with MEFs. The discrepancies between the cell lines may be determined by the metabolic characteristics of the SH-SY5Y as a tumoral cell line [40]. The level of DNA double-strand breaks assessed as *γ*H2AX/53BP1 foci followed the pattern presented by MN accumulation following the exposure to the radiomimetic agent, BLM (Figures 1(d) and 1(e)). The number of foci is higher in both types of cells with PINK1-deficient mitochondria as compared to normal cells. These differences have lower amplitude as compared to the MN induction endpoint. Thus, the data implicates the mitochondrial activity modulated by PINK1 in the cellular vulnerability to genotoxic stress induced by the radiomimetic agent, BLM.

**Figure 2:**
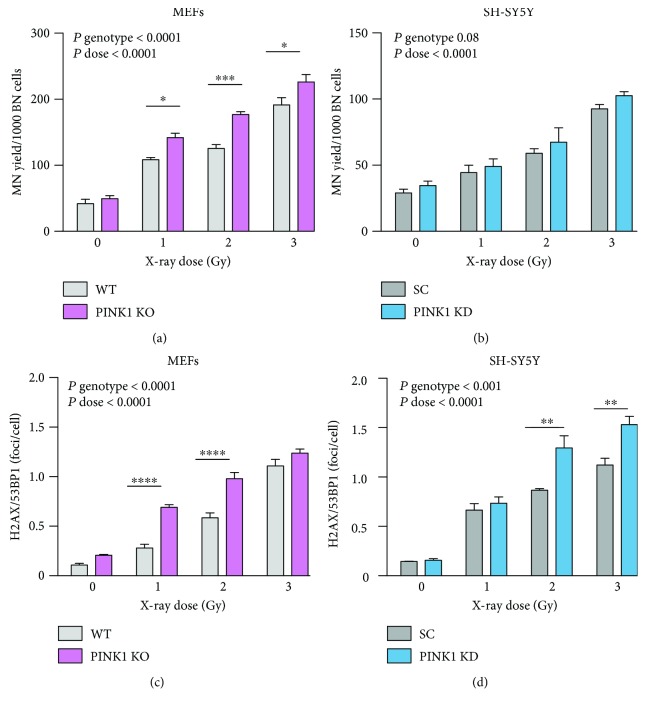
PINK1 loss of function enhances cellular sensitivity to DNA damage induced by X-rays. X-ray irradiation induces the accumulation of DNA damage measured as MN (a, b) and *γ*H2AX/53BP1 foci (c, d) in MEFs (a, c) and SH-SY5Y (b, d). PINK1-KO MEFs and PINK1-KD SH-SY5Y cells present higher sensitivity in the accumulation of both types of DNA damages with the MEFs being more radiosensitive than SH-SY5Y. Each data point represents the mean ± SEM of at least three independent experiments. Statistical analysis is performed with two-way ANOVA with multiple comparisons, and *P* values are indicated in the figure. ^∗^ *P* < 0.05, ^∗∗^ *P* < 0.01, ^∗∗∗^ *P* < 0.001 and ^∗∗∗∗^ *P* < 0.0001.

The data obtained from treating the cells with a different genotoxic agent, X-ray irradiation (Figure 2), reinforces the results obtained for the BLM treatment. Thus, the MN frequency and the number of *γ*H2AX/53BP1 foci increase with the irradiation dose in all the cell types. However, the differences in the MN yield induced in mutant phenotypes as compared to normal ones are less pronounced in both types of cells following irradiation as compared with the BLM treatment (Figures 2(a) and 2(b)). The MEF cells proved again to be more radiosensitive than the SH-SY5Y cells. The analysis of the induction of *γ*H2AX/53BP1 foci by X-ray irradiation (Figures 2(c) and 2(d)) supports the conclusions provided by the MN test. All the effects induced by irradiation have lower amplitude than those induced by BLM treatment.

Taken together, these results indicate that mitochondrial activity modulated by PINK1 is involved in maintaining nuclear DNA integrity.

### 2.2. Additional Measures of Sensitivity to DNA Damage Induced by PINK1 Loss of Function

Besides the measures of DNA damage accumulation, we have investigated the behaviors of other cellular parameters following genotoxic stress to address the role of mitochondria modulated by PINK1 in the maintenance of cellular homeostasis in response to DNA damage induced by BLM. We evaluated the effects of the radiomimetic agent using only the highest concentration employed in our study (40 *μ*g/mL) on viability, induction of apoptosis, accumulation of ROS, and ATP levels in the normal and mutant MEFs.

**Figure 3:**
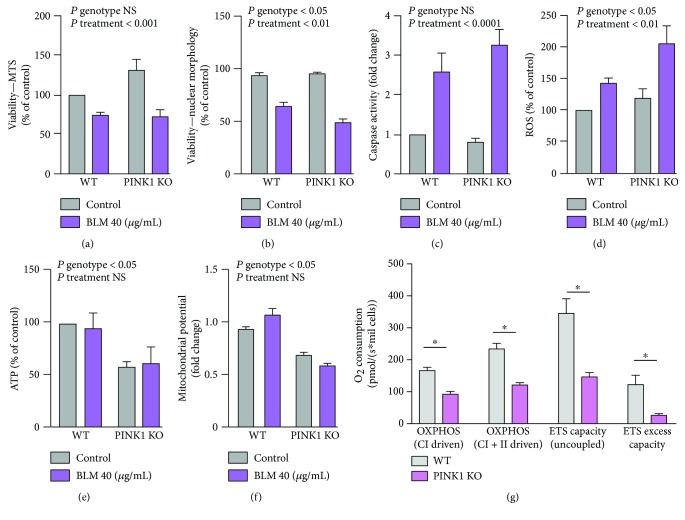
PINK1 role in the intracellular response to genotoxic stress due to BLM. Treatment of MEFs with 40 *μ*g/mL BLM induces decreased viability as assessed by the MTS test and nuclear morphology (a, b), increased activation of caspases as executors of programmed cell death (c), and increased accumulation of ROS (d). The ATP level and mitochondrial potential as measures of mitochondrial function remained unmodified following the treatment with BLM (e, f); however, the basal ATP and mitochondrial levels are diminished in the cells with PINK1 loss of function. The data is reported as percentage of WT untreated control or fold change versus WT untreated control. Each data point represents the mean ± SEM of at least three independent experiments. Statistical analysis is performed with two-way ANOVA with multiple comparisons, and *P* values are indicated in the figure. (g) Analysis of respiratory activity shows that such activity is reduced in the cells with PINK1 loss of function. The respiratory activity was determined using specific substrates and inhibitors for complex I- and II-linked respiration in the presence of ADP. Maximum respiration and ETS were achieved by uncoupling mitochondria with CCCP. ETS excess capacity was calculated by subtracting complex I + II-linked flux from maximum respiration. Each data point represents the mean ± SEM of three independent experiments. Statistical significance was determined with Student's *t*-test. ^∗^ *P* < 0.05.

The bleomycin treatment induced decreased viability of the cells as measured by changes in the nuclear morphology and the MTS assay, with loss of viability being higher in PINK1-deficient cells (Figures 3(a) and 3(b)). Analysis of apoptosis induction (Figure 3(c)) was performed by the measurement of caspase 3/7 level. The level of caspase 3/7 is enhanced (with statistical significance) after the treatment with bleomycin in both types of cells; however, there is no differential activation between WT and PINK1-KO cells. BLM induces an elevation of oxidative stress (Figure 3(d)), this effect being more pronounced in PINK1-deficient cells. This result confirms the involvement of mitochondrial quality control in the cellular stress induced by genotoxic agents.

Mitochondrial function assessed by the measurement of changes in the ATP level and the mitochondrial potential (Figures 3(e) and 3(f)) does not appear to be affected by BLM treatment although both basal ATP levels and mitochondrial potential are decreased in PINK1-KO MEFs as compared to WT. This decrease in ATP production correlates with lower respiratory activity (Figure 3(g)). We have investigated the mitochondrial respiratory activity with high-resolution respirometry using specific substrates for complex I-linked (malate and glutamate) and complex II-linked (Succinate) respiration combined with specific respiratory inhibitors in digitonin-permeabilized cells. The OXPHOS respiratory state as a measure of capacity for oxidative phosphorylation was determined in the presence of ADP while uncoupling for the detection of the electron transfer system (ETS) capacity was achieved with CCCP titration. Interestingly, loss of PINK1 reduces the respiratory activity in all the respiratory parameters followed, that is, coupled respiration of complex I and II (OXPHOS), total uncoupled respiration, and ETS excess capacity. ETS excess capacity is available to drive processes other than phosphorylation without competing with ATP production. This indicates a reduced capacity of PINK1-KO fibroblasts to use mitochondria fully for its metabolic needs.

In SH-SY5Y, the caspase activity and the changes in ATP show the same pattern as in MEF cells. Due to the reduced sensitivity of the cells to the treatment, the amplitude of the change is reduced as compared to the MEFs and other parameters like viability and ROS do not appear to be modified significantly (Supplementary Figure S4).

The data reveals so far exacerbated sensibility to genotoxic stress in PINK1-deficient mammalian cells as revealed by a marked increase in DNA damage correlated with enhanced accumulation of ROS. The lower ATP level and respiratory activity in PINK1-KO cells suggest that PINK1 loss of function does not support the repair processes required following DNA damage leading to increased sensitivity to genotoxic treatments.

**Figure 4:**
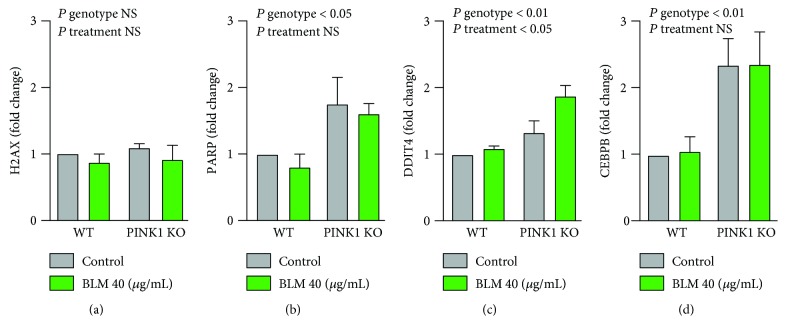
PINK1 role in the transcriptional modulation of some DNA damage/repair factors following genotoxic stress induced by BLM. MEF cells from PINK1-KO and WT control have been treated with BLM at 40 *μ*g/mL for one hour, and the cells were harvested 24 hours later for analysis of changes in transcription factors. DNA damage and repair (a, b) as well as signalling molecules (c, d) were investigated. PINK1 loss of function enhances stress signalling (b, d) while additional genotoxic stress appears to affect only one of the tested stress factors, DDIT4 (c). The data is reported as fold change versus WT untreated control. Each data point represents the mean ± SEM of at least three independent experiments. Statistical analysis was performed with two-way ANOVA with multiple comparisons, and *P* values are indicated in the figure.

We have additionally investigated changes in markers of cellular stress in the experimental conditions presented. Due to the low/medium level of genotoxic insult, BLM did not induce high levels of changes in mitochondrial stress responses, cytoplasmic stress, or ER stress (Supplementary Figure S5). Moreover, there is high variability between experiments with respect to these markers in a context of a low-level genotoxic stress. However, looking at DNA damage and repair factors, we have observed changes reflecting the combined damage induced by mitochondrial function impairment and genotoxic stress (Figure 4). Thus, there is no change at transcriptional level in nonphosphorylated H2AX (Figure 4(a)). However, higher levels in the phosphorylated active form (gamma-H2AX) of the protein are present as shown in Figure 1(d). Significantly, PARP1 is enhanced in PINK1-KO cells reflecting the enhanced need for these cells to deal with constant stress situation generated by PINK1 loss of function (Figure 4(b)) [41, 42]. A similar pattern is presented by CCAAT enhancer protein beta (CEBPB), a bZIP transcription factor implicated in cellular stress responses [43] (Figure 4(d)). Interestingly, these two factors are not modified at transcriptional level by BLM. One particular stress factor that appears to be modified by both PINK1 loss of function and DNA damage is DNA damage-inducible transcript 4 (DDIT4) (Figure 4(c)). DDIT4 regulates cell growth, proliferation, and survival via the mammalian target of rapamycin complex 1 (mTORC1). Most significantly, DDIT4 has been shown to change its levels in response to stress and to be enhanced in brains with age-related neurodegenerative conditions like Parkinson's disease [44, 45].

### 2.3. PINK1 Loss of Function Contributes to the Induction of Adaptive Response

The low-dose-induced adaptive response by genotoxic agents represents a unique communication path between DNA and cellular metabolism. Here, we have tested whether PINK1 loss of function may induce cellular changes that make the cells more prone to respond to DNA damage induced by BLM in an adaptive manner.

**Figure 5:**
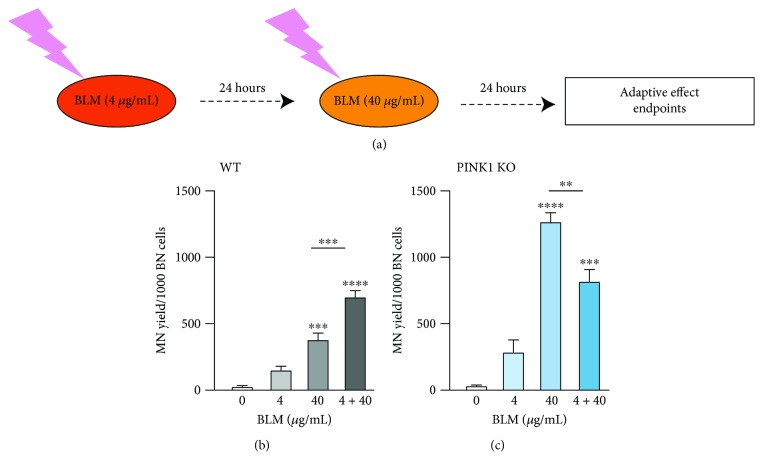
PINK1 loss of function leads to the induction of an adaptive response by BLM in MEFs. (a) Design of adaptive response experiments. The cells were exposed to low-dose genotoxic treatment (4 *μ*g/mL BLM) and incubated for 24 hours with fresh medium. The cells were treated again with a higher dose of BLM (40 *μ*g/mL). The genotoxicity was analyzed 24 h later for all the treatments 4 *μ*g/mL, 40 *μ*g/mL, and 4 + 40 *μ*g/mL. A reduction in genotoxicity at the combined treatment of 4 + 40 *μ*g/mL compared to the 40 *μ*g/mL treatment signifies an adaptive response. (b, c) WT MEFs do not respond adaptively while the PINK1-KO MEFs show an adaptive response following these treatments. Each data point represents the mean ± SEM of at least three independent experiments. Statistical analysis is performed with one-way ANOVA with multiple comparisons, and the significance level is indicated in the figure. ^∗∗^ *P* < 0.01, ^∗∗∗^ *P* < 0.001 and ^∗∗∗∗^ *P* < 0.0001.

In WT and KO MEFs, 4 *μ*g/mL BLM was used as a priming dose and 40 *μ*g/mL BLM was used as a challenging dose, with a 24 h interval between treatments (Figure 5(a)). The MN frequency was detected to observe the adaptive response induced by the low dose of BLM. The MN yield increased significantly by the treatment with the challenging dose in both WT and PINK1-KO cells with higher amplitude in PINK1-KO MEFs as it was expected following the results presented previously. However, a priming dose of BLM treatment attenuated the elevation of MN frequency following the additional treatment with the challenging dose in PINK1-KO MEFs but not in WT MEFs (Figures 5(b) and 5(c)). The adaptive response was observed only for MEFs as the neuronal SH-SY5Y cells did not respond adaptively (data not shown).

Our results suggest that mitochondrial activity plays a role in adjusting cellular function to cope with detrimental stress, depending on the cell type.

### 2.4. PINK1 Loss of Function Impairs Transmission of Bystander Signalling

Recent evidence shows that apart from the intracellular signalling, the directly damaged cells send intercellular signals to their neighbour bystander cells.

**Figure 6:**
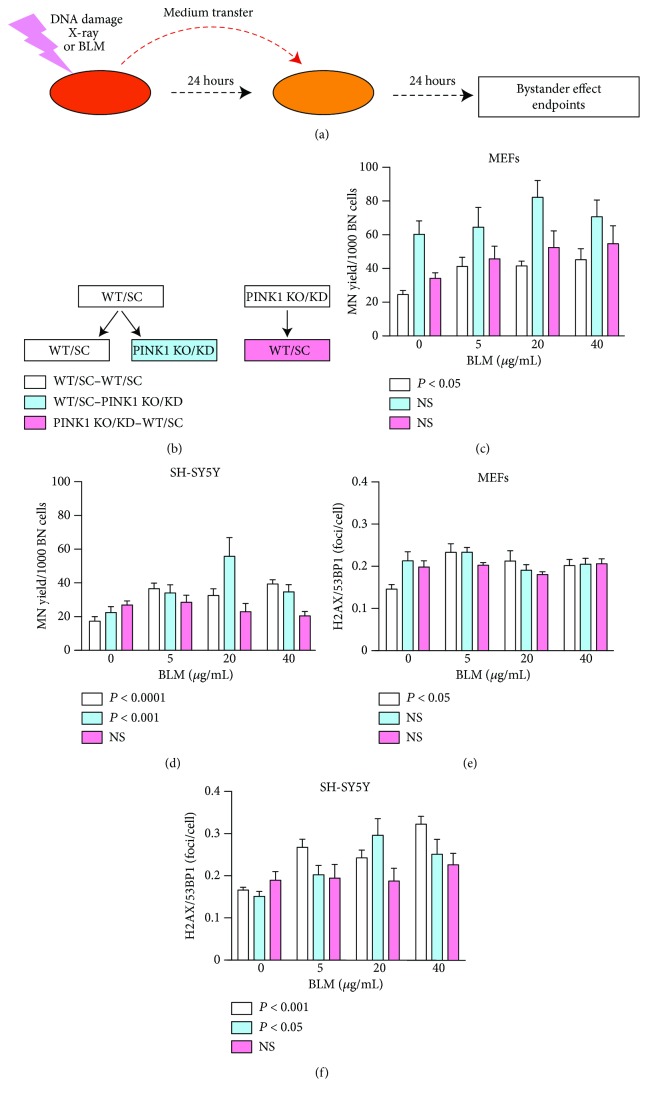
PINK1 loss of function impairs transmission of intercellular, bystander signalling. (a, b) Experimental scheme for the study of the bystander effect. For the direct treatment, the cells were exposed to BLM for 1 hour and then washed and incubated with fresh medium. This medium was conditioned for 24 hours with bystander factors by the directly treated cells and was used for the study of the bystander effect. The bystander cells are grown in medium transferred from BLM-treated donor cells collected at 24 h posttreatment. The transfer of media was performed between WT and KO MEFs and SC and KD SH-SY5Y as follows. The medium was transferred either from WT/SC to WT/SC and WT/SC to PINK1 KO/KD or from PINK1-KO/KD to WT/SC cells. (c, e) In MEFs, the MN induction and the number of *γ*H2AX/53BP1 foci increase with the BLM concentration only when the WT cells are grown 24 h in medium transferred from WT cells. (d, f) For SH-SY5Y, the MN yield and *γ*H2AX/53BP1 foci accumulated in both bystander SC and PINK1-KD cells grown in medium transferred from SC NB cells. Each data point represents the mean ± SEM of at least three independent experiments. *P* values from one-way ANOVA are indicated in the figure.

We investigated the relationship between mitochondrial function integrity modulated by PINK1 and bystander signalling induced by BLM. We adopted a medium transfer technology with the medium being transferred to the receiver cells (bystander) from the treated donor cells incubated 24 h with the medium (Figure 6(a)). We studied the cellular behaviour in the medium transfer either from WT/SC to WT/SC and WT/SC to PINK1-KO/KD or from PINK1-KO/KD to WT/SC cells. After the medium transfer, the bystander cells are grown in this medium for another 24 h prior to analysis (Figure 6(b)).

In Figure 6(c), we show that the MN yield presents a significant increase in the WT MEFs treated with bystander medium from WT cells. Conditioned medium from WT cells treated by BLM induced bystander responses by increasing genotoxicity in WT and PINK-KD SH-SY5Y cells (Figure 6(d)). Medium from PINK1 KO/KD was not able to induce these types of effects.

A similar trend is observed by analyzing double-strand breaks measured as *γ*H2AX/53BP1 foci in MEFs (WT and PINK1 KO) and SH-SY5Y (SC and PINK1 KD) treated with bystander medium (Figures 6(e) and 6(f)). This parameter shows a significant increase in the WT MEFs and SC SH-SY5Y cells and also in PINK-KD SH-SY5Y cells receiving medium from WT MEFs and SC SH-SY5Y, respectively. No bystander response could be observed in WT MEFs and SC SH-SY5Y cells receiving conditioned medium from PINK1-KO MEFs or PINK-KD SH-SY5Y cells, respectively.

The level of DNA damage (revealed by both parameters of MN and *γ*H2AX/53BP1 foci) in bystander cells is lower than that induced by direct BLM treatment and is not dependent on the increase of the treatment dose. This phenomenon is common in bystander effect signalling, and it is known as “saturation response” [46]. We have obtained the same pattern of bystander response in MEFs and SH-SY5Y using the primary genotoxic agent the X-ray irradiation (Supplementary Figure S6).

These findings revealed, for the first time, the important role of mitochondrial function modulated by PINK1 in transmitting the intercellular, bystander signalling following DNA damage.

## 3. Discussion

Increasing evidence shows that mitochondrial dysfunction is associated with age-related diseases. The PINK1 gene encodes a highly conserved serine-threonine kinase mutations which cause autosomal recessive Parkinsonism. These mutations compromise the kinase activity or interfere with protein stability suggesting a loss-of-function mechanism in PD. The effect of PINK1 loss of function on Parkinson's disease models has been thoroughly documented and has highlighted the role of PINK1 in the maintenance of mitochondrial quality control and cellular homeostasis. Thus, PINK1 has been shown to protect against cell death induced by various toxins, while PINK1 depletion increases vulnerability to oxidative stress-induced cell death. Several mechanisms have been proposed to explain the cytoprotective activity of PINK1, including effects on mitochondrial bioenergetics and calcium homeostasis. Thus, through the study of Parkinson's disease-related etiopathology, a great deal of information related to the role of PINK1 in the maintenance of cellular homeostasis has been generated and the information has been related to a wide range of age-related dysfunctions (reviewed in [47]).

**Figure 7:**
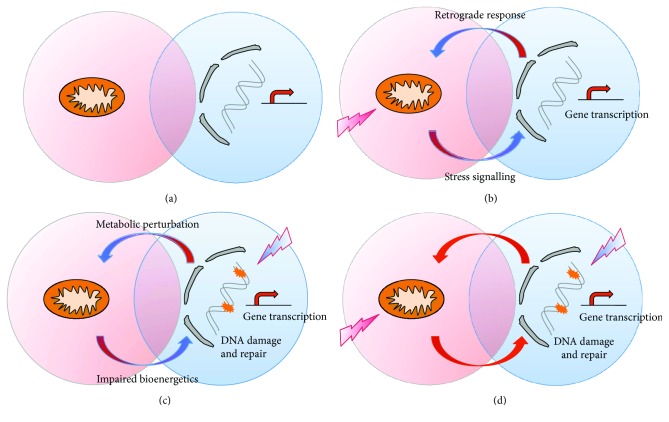
The model of mitochondria-nucleus communication in the maintenance of cellular homeostasis during combined mitochondrial and genotoxic stress. Mitochondria and the nucleus communicate to maintain cellular homeostasis. Mitochondria produce ATP and metabolites required for nuclear activity, while the nucleus is responsible for encoding most mitochondrial proteins (a). Following mitochondrial stress, mitochondria accumulate damage that leads to increased ROS and altered ATP production. These trigger the signalling to the nucleus that is resulting in a retrograde response consisting of transcriptional and metabolic responses designed to reestablish the cellular homeostasis (b). Genotoxic damage impacts the mitochondria primarily affecting mitochondrial protein synthesis and ATP production and enhancing oxidative stress (c). Combined stresses at the level of mitochondria and the nucleus likely to appear in aging and age-related disease impair both mitochondrial processes (e.g., mtQC) and nucleus function (e.g., DNA repair). Here, we provide examples of how cumulative genotoxic stress (DNA damage) and mitochondrial dysfunction (PINK1 loss of function) are contributing to changes in cellular homeostasis and intercellular communication (d).

Our study revealed new findings which emphasize that mitochondria through PINK1 have an important contribution to mitochondria-nucleus communication for the maintenance of cell homeostasis under external cellular genotoxic stress conditions. The proposed model for mitochondria-nucleus communication is depicted in Figure 7. Mitochondria and the nucleus communicate normally in maintaining cellular homeostasis by mitochondria producing ATP and metabolites required for nuclear activity. In turn, the nucleus is responsible for encoding most mitochondrial proteins (Figure 7(a)). Following mitochondrial stress which in our model is achieved by PINK1 loss of function, mitochondria accumulate damage that leads to increased ROS and altered ATP production. All these trigger the signalling to the nucleus that is resulting in a retrograde response consisting of transcriptional changes, metabolic adaptations, enhanced mtQC, and decreased ROS in order to reestablish the cellular homeostasis (Figure 7(b)). The mechanisms of mitochondrial stress signalling have been recently reviewed [2]. Accumulation of DNA damage also impacts the mitochondrial function via multiple mechanisms: DNA damage is associated with enhanced oxidative stress. DNA repair enzymes use NAD+ thus reducing the availability of substrates for mitochondrial function; as a consequence, synthesis of mitochondrial proteins may be affected (Figure 7(c)). Recent evidence supports the hypothesis that mitochondrial abnormalities appear to be enhanced by the accumulation of DNA damage via decreased activation of the NAD+-SIRT1-PGC1a pathway triggered by hyperactivation of the DNA damage sensor PARP1 [41, 42].

Combined stresses at the level of mitochondria and the nucleus which are likely to appear in neurodegenerative diseases/aging impair both mitochondrial processes (e.g., mtQC) and nuclear function (e.g., DNA repair), and in the current study, we have provided examples of increased accumulation of DNA damage upon PINK1 loss of function, adaptive response in PINK1-KO cells, and impaired bystander signalling (Figure 7(d)). An interesting feature that has characterized the mitochondria-nucleus communication in our experimental conditions is that on a background of PINK1 loss of function, DNA damage triggered with BLM did not induce additional mitochondrial damage as reflected by the lack of change in the mitochondrial function parameters measured here. This could also be determined by the way in which the experiment is designed; that is, the treatment lasts for one hour and the endpoints are measured 24 hours later. During this time, potentially many of the cells that did not accumulate damage would proliferate and compensate in the overall measurement. We have used this experimental approach to correspond with the study of the bystander effects where the media need to be conditioned for a time interval (in our model, 24 hours) after the direct treatment. Overall, in the combined mitochondrial and DNA stress, DNA repair mechanisms appear to be a priority in order to maintain cellular homeostasis.

Stress insults were triggered by a BE in WT cells but not by an adaptive response. It was suggested that radiation or chemically induced bystander effects reflect the function of intercellular communication. This is considered a mechanism by which DNA lesions that accumulated in hit cells are transmitted to other cells and spread within tissues or even the whole organism [48]. These responses might be protective at the organism level by enhancing repair in a community of cells and by eliminating severely damaged cells. PINK1-deficient cells showed altered intercellular signalling of stress, impairing the induction of bystander phenomena, by suppressing signal formation in treated cells and also by altering the capacity to respond to the signals in neighboring cells. We hypothesize that directly treated PINK1-deficient cells have a reduced ability to release bystander factors in the extracellular environment potentially due to reduced energetic capacity. When PINK1-deficient cells are at the receiving end of the bystander signal, they also present impaired ability of processing the bystander signalling depending on the cell type. Thus, only in SH-SY5Y, PINK1-KD cells respond to the bystander signalling transmitted by SC cells. Although PINK1-deficient cells present impaired bystander signalling, they respond adaptively when a challenge dose (BLM 40 *μ*g/mL) is applied subsequently to a low-dose treatment (BLM 4 *μ*g/mL) to the cells. The low dose increased slightly the existent basal stress in mitochondria and triggered a stimulation of DNA repair processes, and consequently, the cells appear to cope better with the challenge dose. This phenomenon could be due to the mitohormesis ability of mitochondria which are exposed to a background level of endogenous stress. The augmented stress resistance of mitochondria to subsequent stress is well known [49]. Interestingly, the WT cells did not present this adaptive response. As the accumulation of DNA damage is reduced as compared to the damage accumulated in the KO cells, we hypothesize that the stress was not sufficiently challenging in the WT cells to trigger the adaptive mechanisms.

In our model, oxidative stress that results from both mitochondrial dysfunction and DNA damage appears to be a common mediator of cellular damage, but it is also demonstrating a key role in the generation and transmission of signals either at intracellular or at intercellular level. This dual role of oxidative species is reviewed by Pizzino et al. [50]. These oxidative species may mediate adaptive responses or genomic instability in the progeny of both directly and bystander stressed cells depending on their concentration, reactivity, and spatial and temporal distribution [51].

Cellular defenses against the oxidative stress have adapted to limit the dangerous effects of ROS while maintaining their signalling capacity. Therefore, cells evolved different mechanisms of response to protect the cells facing the oxidative challenge generated by ROS. The cellular stress responses at low doses are likely to be a complex interplay among direct effects, the adaptive response, and the bystander effect.

Our hypothesis is that PINK1 contributes to the management of cellular stress being involved in bystander transmission of signals through intercellular communication. Thus, we demonstrate for the first time the involvement of the PINK1 kinase not only in intracellular signalling but also in intercellular signalling. In our study, AR and BE represent two facets of the cell-protective mechanisms against stress insults. These results point to an important mitochondrial contribution to cellular homeostasis preventing the accumulation of detrimental effects through PINK1 kinase functions.

## 4. Conclusion

Our study demonstrates that mitochondrial activity modulated by PINK1 is involved in cellular responses to genotoxic stress. Thus, mitochondrial function modulated by PINK1 contributes to the management of cellular stress by intracellular response preventing the accumulation of detrimental effects. Moreover, in the process of intercellular communication following DNA lesions, PINK1 loss-of-function cells show an impaired bystander signalling but respond adaptively to a challenge dose, probably due to similar mechanisms involved in mitohormesis.

Taken together, all these results pinpointed to the role of PINK1 in the intimate connection between mitochondrial dysfunction and nuclear-dependent retrograde response.

## 5. Materials and Methods

### 5.1. Cell Culture

The experiments were done on wild-type (WT) or PINK1-knockout (KO) mouse embryonic fibroblasts (MEFs), a gift from Dr. LM Martins, and WT and PINK1-knockdown (KD) human neuroblastoma SH-SY5Y, a gift from Dr. H Plun-Favreau. Cells were used until passage 25 in a humidified incubator at 37°C. MEFs were grown in DMEM supplemented with 10% FBS, 2 mM L-glutamine, 50 U/mL penicillin, and 50 *μ*g/mL streptomycin, and SH-SY5Y cells in DMEM/F12 with the same supplements.

### 5.2. Genotoxic Treatment, Medium Transfer, and Adaptive Induction

Cells were directly exposed to 0–3 Gy of X-ray generated by a medical linear accelerator (Primus Mevatron 2D, 6 MV, Siemens, Germany), at a dose rate of 1.85 Gy/min [23], or treated with bleomycin sulfate (BLM, Sigma-Aldrich, USA) for 1 hour at 5–40 *μ*g/mL followed by 3 washes with culture medium [22].

Bystander effects were induced by the medium transfer method. Following genotoxic treatment, the cells (directly treated, donor cells) were incubated for 24 h in fresh medium in order to allow secretion of bystander factors. This medium (bystander conditioned) was then collected, filtered (0.22 *μ*m filters, Millipore, Germany), and transferred onto a new lot of cells. The untreated cells (bystander, receiver cells) are grown in medium transferred from treated donor cells collected at 24 h posttreatment. The transfer of media was performed between WT and KO MEFs and SC and KD SH-SY5Y as follows. The medium was transferred either from WT/SC to WT/SC and WT/SC to PINK1-KO/KD or from PINK1-KO/KD to WT/SC cells.

For cellular adaptive response induction, the cells were treated with a low concentration of BLM, 4 *μ*g/mL for 1 hour, washed 3 times with culture medium, and then added with fresh medium. We incubated the cells for additional 24 hours; then, we treated the cells with a high concentration of BLM (40 *μ*g/mL) for 1 hour, followed by 3 washes with culture medium and addition of fresh medium. In each experiment, we used a negative control (no BLM treatment), a low-dose control (treated only with the first 4 *μ*g/mL BLM), and a high-dose control (treated only with 40 *μ*g/mL BLM) in addition to the sample that was exposed to the combined treatment with 4 *μ*g/mL and 40 *μ*g/mL.

### 5.3. Analysis of *γ*-H2AX/53BP1 Foci

Staining of foci was performed by a classic immunofluorescence protocol. The cells were fixed in 3.7% paraformaldehyde (Sigma, Germany) for 20 minutes, permeabilized in 0.1% Triton-X (Sigma, Germany) for 20 minutes, and blocked with 5% donkey serum (Jackson ImmunoResearch, USA) for 1 hour. We used primary antibodies rabbit anti-53BP1 (Abcam, UK; 1 : 800) and mouse anti-*γ*-H2AX (Millipore, Germany; 1 : 400) overnight at 4°C and secondary antibodies donkey anti-rabbit conjugated with Rhodamine Red (Jackson ImmunoResearch, USA; 1 : 200) and donkey anti-mouse conjugated with Alexa Fluor 488 (Jackson ImmunoResearch, USA; 1 : 200) for 1 hour at room temperature [52]. The images were taken with a fluorescence microscope, and the analysis was performed by manual scoring. For scoring of gamma-H2AX, slides were visualized using a fluorescence microscope (Olympus BX51) equipped with appropriate filters for DAPI, Alexa 488, and Rhodamine Red and with 100x objective. Representative images for the conditions employed in this study are presented in Supplementary Figure S2. For each sample, foci were scored in 100 nuclei. The nuclei were identified by DAPI staining. In each nucleus, we counted foci represented by an intense staining with both gamma-H2AX and 53BP1 markers. Cells with condensed nuclei and saturated staining were not scored, as these are markers of apoptosis. We calculated the average number of foci per cell for each condition. In each experiment, we scored 3 slides for each experimental point. The results represent mean ± SEM for 3 independent experiments.

### 5.4. Micronuclei Analysis

The micronuclei assay protocol used was adapted from [51] for using fluorescence staining. Cells were seeded on glass coverslips and treated as described (BLM, X-ray, or bystander medium). Cytochalasin B was added 20 h before analysis at 3 *μ*g/mL to MEF cells and 6 *μ*g/mL to SH-SY5Y. For staining, cells were washed in PBS, fixed in acetic acid : methanol (1 : 9), and stained with Acridine Orange at 10 *μ*g/mL for 10 minutes followed by washing in PBS and mounting [23]. Representative images for the conditions employed in this study are presented in Supplementary Figure S3. Micronuclei were scored according to the Fenech criteria in 1000 binucleated cells [53].

### 5.5. Cellular Viability—MTS Test

The cellular viability was determined by the MTS test, a colorimetric method based on mitochondrial oxidoreductase activity according to the manufacturer's instructions (G3581, Promega). Briefly, the medium was removed, and MTS solution was added at a ratio of 1 : 5 in culture medium with 5% FBS. This was incubated for 3 h, and the absorbance was measured at 490 nm using a spectrophotometer (Mithras, Berthold Technologies). In each experiment, we used at least 3 technical replicates per biological condition and the corresponding negative controls and blank samples.

### 5.6. Cellular Viability—Nuclear Morphology

At the time of analysis, cells were first washed once with PBS, fixed in 3.7% PFA for 20 minutes, washed 3 times in PBS, and permeabilized with 0.1% Triton-X for another 20 minutes. Staining was done by incubation with a solution of 1 *μ*g/mL of Hoechst 33342 (Thermo Fisher, USA) in PBS for 30 minutes, followed by washing 3 times with PBS.

The cells were visualized using fluorescence microscopy. Images corresponding to at least 500 cells were taken for each sample. The cells were analyzed manually by the following criteria: cells with round or oval nuclei and with regular shape and color were counted as live and cells with condensed and small nuclei and with irregular shape or nuclear fragmentation were scored as apoptotic. At least 500 cells per sample were analyzed in 3 independent experiments.

### 5.7. Mitochondrial Potential

The changes in mitochondrial potential were measured with TMRE (Invitrogen) according to the manufacturer's protocols. Briefly, the cells were plated in 24-well plates and treated as described for the direct effect. At the time of the analysis, they were loaded with 100 nM TMRE in the culture medium for 30 min; then, the cells were analyzed by flow cytometry (Accuri C6, BD Bioscience). The relative difference in mitochondrial potential was determined by the relative change in the percentage of cells gated in the higher fluorescence intensity window, which was set for the control cells at about 10%.

### 5.8. ROS Measurement

H_2_O_2_ was measured using the ROS-Glo Assay Kit (Promega) following the manufacturer's instructions (G8820, Promega). The cells were seeded in 96-well plates. Three hours before the analysis, the medium was removed and 100 *μ*L of a solution of 25 *μ*M H_2_O_2_ substrate was added. For analysis, 100 *μ*L of ROS-Glo solution was added, followed by 20-minute incubation at room temperature. The chemiluminescence was measured using a spectrophotometer (Mithras, Berthold Technologies).

### 5.9. Caspase 3/7 Analysis

Apoptosis induction was evaluated by changes in caspase 3/7 levels, determined by a chemiluminescence method according to the manufacturer's protocol (G8090, Promega). The caspase levels were normalized to protein content in each sample.

### 5.10. ATP Level Measurement

ATP levels were determined using CellTiter-Glo (Promega) following the manufacturer's instructions (G7572). Briefly, the cells were grown in 96-well plates in 100 *μ*L medium. For analysis, we added the mix provided by the kit to the cells and medium, incubated for 30 minutes, and the chemiluminescence was measured using a spectrophotometer (Mithras, Berthold Technologies). ATP levels were quantified using ATP standard curves, by serial dilution of ATP (Sigma, Germany) in culture medium. Furthermore, the ATP levels were normalized to protein content, measured by the Bradford assay.

### 5.11. Protein Level Measurement (Bradford Method)

Protein levels were measured for normalization purposes using the Bradford method. Briefly, the cells grown in 96-well plates were washed in PBS, permeabilized with 1 : 7 acetic acid : methanol for 20 minutes, and incubated with the Bradford solution (Sigma, Germany) for 30 minutes. Cells were then washed with PBS, and we added ethanol for solubilisation of staining complexes. In each experiment, we normalized the samples to a standard curve prepared by a BSA standard stock solution (in PBS) with serial dilutions made in the Bradford solution. Each experiment included blank samples for test samples, standard curve, and negative controls.

### 5.12. Gene Expression Analysis

RNA was extracted using TRIzol (Sigma) according to the manufacturer's instructions. Reverse transcription was performed using the High-Capacity Reverse Transcription Kit (Applied Biosystems). Quantitative RT-PCR was performed with an Applied Biosystems cycler using the SYBR Green RT-PCR system. Gene-specific primers were designed and obtained from Sigma. The relative transcript levels of the target genes were normalized against *GAPDH* mRNA levels. Quantification was performed using the comparative Ct method [54].

### 5.13. Statistical Analyses

Data are presented as mean values, and error bars indicate ±SD or ±SEM as noted. Inferential statistical analysis was performed using the Prism and StatMate software packages (http://www.graphpad.com). The significance level is indicated as ^∗∗∗∗^ *P* < 0.0001, ^∗∗∗^ *P* < 0.001, ^∗∗^ *P* < 0.01, and ^∗^ *P* < 0.05, and NS indicates *P* > 0.05.

### 5.14. Measurement of Respiratory Activity

Mitochondrial respiration was assayed at 37°C by high-resolution respirometry using an OROBOROS Oxygraph. The DatLab software package (OROBOROS, Innsbruck, Austria) was used for data acquisition and analysis. Complex I- and complex I- and II- driven activities were assayed in MiR05 respiration buffer (20 mM HEPES, 10 mM KH_2_PO_4_, 110 mM sucrose, 20 mM taurine, and 60 mM K-lactobionate), 5 mM EGTA, 3 mM MgCl_2_ × 6H_2_O, and 1 g/L BSA (fatty acid free) in the presence of saturating ADP (5–10 mM)) and using the substrates malate (2 mM), glutamate (10 mM), and succinate (10 mM). Uncoupling was achieved with CCCP titrations.

## Data Availability

The data used to support the findings of this study are available from the corresponding author upon request.

## Supplementary Materials

Supplementary Materials The Supplementary File contains six supplementary figures. Supplementary Figure 1: level of PINK1 downregulation in the cell lines used in the study and the effect of PINK1 loss of function on basal ATP level. Supplementary Figure 2: representative images of *γ*-H2AX/53BP1 staining. Supplementary Figure 3: representative images of micronuclear staining. Supplementary Figure 4: impact of BLM treatment on SH-SY5Y viability, caspase activation, oxidative species accumulation, and ATP. Supplementary Figure 5: compartmental stress signalling does not appear to be affected by BLM. Supplementary Figure 6: PINK1 loss of function impairs transmission of intercellular, bystander signalling following X-ray irradiation.

## References

[1] Quirós P. M., Mottis A., Auwerx J. (2016). Mitonuclear communication in homeostasis and stress. *Nature Reviews Molecular Cell Biology*.

[2] Shpilka T., Haynes C. M. (2018). The mitochondrial UPR: mechanisms, physiological functions and implications in ageing. *Nature Reviews Molecular Cell Biology*.

[3] Pickles S., Vigie P., Youle R. J. (2018). Mitophagy and quality control mechanisms in mitochondrial maintenance. *Current Biology*.

[4] Yamano K., Youle R. J. (2013). PINK1 is degraded through the N-end rule pathway. *Autophagy*.

[5] Greene A. W., Grenier K., Aguileta M. A. (2012). Mitochondrial processing peptidase regulates PINK1 processing, import and Parkin recruitment. *EMBO Reports*.

[6] Kane L. A., Lazarou M., Fogel A. I. (2014). PINK1 phosphorylates ubiquitin to activate Parkin E3 ubiquitin ligase activity. *Journal of Cell Biology*.

[7] Kazlauskaite A., Kondapalli C., Gourlay R. (2014). Parkin is activated by PINK1-dependent phosphorylation of ubiquitin at Ser^65^. *Biochemical Journal*.

[8] Koyano F., Okatsu K., Kosako H. (2014). Ubiquitin is phosphorylated by PINK1 to activate Parkin. *Nature*.

[9] Valente E. M., Abou-Sleiman P. M., Caputo V. (2004). Hereditary early-onset Parkinson’s disease caused by mutations in *PINK1*. *Science*.

[10] Matsuda S., Kitagishi Y., Kobayashi M. (2013). Function and characteristics of PINK1 in mitochondria. *Oxidative Medicine and Cellular Longevity*.

[11] Celardo I., Costa A. C., Lehmann S. (2016). Mitofusin-mediated ER stress triggers neurodegeneration in *pink1/Parkin* models of Parkinson’s disease. *Cell Death & Disease*.

[12] Glorioso C., Oh S., Douillard G. G., Sibille E. (2011). Brain molecular aging, promotion of neurological disease and modulation by Sirtuin5 longevity gene polymorphism. *Neurobiology of Disease*.

[13] O'Flanagan C. H., Morais V. A., Wurst W., De Strooper B., O'Neill C. (2015). The Parkinson’s gene PINK1 regulates cell cycle progression and promotes cancer-associated phenotypes. *Oncogene*.

[14] Jackson S. P., Bartek J. (2009). The DNA-damage response in human biology and disease. *Nature*.

[15] Ribezzo F., Shiloh Y., Schumacher B. (2016). Systemic DNA damage responses in aging and diseases. *Seminars in Cancer Biology*.

[16] Roos W. P., Thomas A. D., Kaina B. (2016). DNA damage and the balance between survival and death in cancer biology. *Nature Reviews Cancer*.

[17] Halazonetis T. D., Gorgoulis V. G., Bartek J. (2008). An oncogene-induced DNA damage model for cancer development. *Science*.

[18] Dimova E. G., Bryant P. E., Chankova S. G. (2008). Adaptive response: some underlying mechanisms and open questions. *Genetics and Molecular Biology*.

[19] Widel M., Krzywon A., Gajda K., Skonieczna M., Rzeszowska-Wolny J. (2014). Induction of bystander effects by UVA, UVB, and UVC radiation in human fibroblasts and the implication of reactive oxygen species. *Free Radical Biology & Medicine*.

[20] Hei T. K., Zhou H., Chai Y., Ponnaiya B., Ivanov V. N. (2011). Radiation induced nontargeted response: mechanism and potential clinical implications. *Current Molecular Pharmacology*.

[21] Chevalier F., Hamdi D. H., Saintigny Y., Lefaix J. L. (2015). Proteomic overview and perspectives of the radiation-induced bystander effects. *Mutation research/Reviews in mutation research*.

[22] Savu D., Petcu I., Temelie M., Musctaciosu C., Moisoi N. (2015). Compartmental stress responses correlate with cell survival in bystander effects induced by the DNA damage agent, bleomycin. *Mutation Research/Fundamental and Molecular Mechanisms of Mutagenesis*.

[23] Temelie M., Stroe D., Petcu I., Mustaciosu C., Moisoi N., Savu D. (2016). Bystander effects and compartmental stress response to X-ray irradiation in L929 cells. *Radiation and Environmental Biophysics*.

[24] Shao C., Aoki M., Furusawa Y. (2003). Bystander effect on cell growth stimulation in neoplastic HSGc cells induced by heavy-ion irradiation. *Radiation and Environmental Biophysics*.

[25] Sprung C. N., Ivashkevich A., Forrester H. B., Redon C. E., Georgakilas A., Martin O. A. (2015). Oxidative DNA damage caused by inflammation may link to stress-induced nontargeted effects. *Cancer Letters*.

[26] Barcellos-Hoff M. H., Brooks A. L. (2001). Extracellular signaling through the microenvironment: a hypothesis relating carcinogenesis, bystander effects, and genomic instability. *Radiation Research*.

[27] Kim C. S., Kim J. M., Nam S. Y. (2007). Low-dose of ionizing radiation enhances cell proliferation via transient ERK1/2 and p38 activation in normal human lung fibroblasts. *Journal of Radiation Research*.

[28] Kim C. S., Kim J. K., Nam S. Y. (2007). Low-dose radiation stimulates the proliferation of normal human lung fibroblasts via a transient activation of Raf and Akt. *Molecules & Cells*.

[29] Shimura T., Kunugita N. (2016). Mitochondrial reactive oxygen species-mediated genomic instability in low-dose irradiated human cells through nuclear retention of cyclin D1. *Cell Cycle*.

[30] López-Otín C., Blasco M. A., Partridge L., Serrano M., Kroemer G. (2013). The hallmarks of aging. *Cell*.

[31] Moskalev A., Plyusnina E., Shaposhnikov M., Shilova L., Kazachenok A., Zhavoronkov A. (2012). The role of D-GADD45 in oxidative, thermal and genotoxic stress resistance. *Cell Cycle*.

[32] Madabhushi R., Pan L., Tsai L. H. (2014). DNA damage and its links to neurodegeneration. *Neuron*.

[33] Chow H. M., Herrup K. (2015). Genomic integrity and the ageing brain. *Nature Reviews Neuroscience*.

[34] Zhang J. (2017). Brothers in arms: emerging roles of RNA epigenetics in DNA damage repair. *Cell & Bioscience*.

[35] Mavragani I. V., Nikitaki Z., Souli M. P. (2017). Complex DNA damage: a route to radiation-induced genomic instability and carcinogenesis. *Cancer*.

[36] Qin X., Zheng C., Yates J. R., Liao L. (2014). Quantitative phosphoproteomic profiling of PINK1-deficient cells identifies phosphorylation changes in nuclear proteins. *Molecular BioSystems*.

[37] Moisoi N., Fedele V., Edwards J., Martins L. M. (2014). Loss of *PINK1* enhances neurodegeneration in a mouse model of Parkinson’s disease triggered by mitochondrial stress. *Neuropharmacology*.

[38] Wood-Kaczmar A., Gandhi S., Yao Z. (2008). PINK1 is necessary for long term survival and mitochondrial function in human dopaminergic neurons. *PLoS One*.

[39] Committee to Assess Health Risks from Exposure to Low Levels of Ionizing Radiation, National Research Council (2006). *Health Risks from Exposure to Low Levels of Ionizing Radiation BEIR VII Phase 2*.

[40] Ross R. A., Spengler B. A., Biedler J. L. (1983). Coordinate morphological and biochemical interconversion of human neuroblastoma cells. *Journal of the National Cancer Institute*.

[41] Fang E. F., Scheibye-Knudsen M., Brace L. E. (2014). Defective mitophagy in XPA via PARP-1 hyperactivation and NAD^+^/SIRT1 reduction. *Cell*.

[42] Lehmann S., Costa A. C., Celardo I., Loh S. H. Y., Martins L. M. (2016). *Parp* mutations protect against mitochondrial dysfunction and neurodegeneration in a PARKIN model of Parkinson’s disease. *Cell Death & Disease*.

[43] Zhao Q., Wang J., Levichkin I. V., Stasinopoulos S., Ryan M. T., Hoogenraad N. J. (2002). A mitochondrial specific stress response in mammalian cells. *The EMBO Journal*.

[44] Malagelada C., Ryu E. J., Biswas S. C., Jackson-Lewis V., Greene L. A. (2006). RTP801 is elevated in Parkinson brain substantia nigral neurons and mediates death in cellular models of Parkinson’s disease by a mechanism involving mammalian target of rapamycin inactivation. *The Journal of Neuroscience*.

[45] Canal M., Martín-Flores N., Pérez-Sisqués L. (2016). Loss of NEDD4 contributes to RTP801 elevation and neuron toxicity: implications for Parkinson’s disease. *Oncotarget*.

[46] Hamada N., Matsumoto H., Hara T., Kobayashi Y. (2007). Intercellular and intracellular signaling pathways mediating ionizing radiation-induced bystander effects. *Journal of Radiation Research*.

[47] Voigt A., Berlemann L. A., Winklhofer K. F. (2016). The mitochondrial kinase PINK1: functions beyond mitophagy. *Journal of Neurochemistry*.

[48] Klammer H., Mladenov E., Li F., Iliakis G. (2015). Bystander effects as manifestation of intercellular communication of DNA damage and of the cellular oxidative status. *Cancer Letters*.

[49] Yun J., Finkel T. (2014). Mitohormesis. *Cell Metabolism*.

[50] Pizzino G., Irrera N., Cucinotta M. (2017). Oxidative stress: harms and benefits for human health. *Oxidative Medicine and Cellular Longevity*.

[51] Azzam E. I., Jay-Gerin J. P., Pain D. (2012). Ionizing radiation-induced metabolic oxidative stress and prolonged cell injury. *Cancer Letters*.

[52] Temelie M., Mustaciosu C., Flonta M. L., Savu D. (2017). Cellular differentiation exacerbates radiation sensitivity *in vitro* in a human dopaminergic neuronal model. *Romanian Reports in Physics*.

[53] Fenech M. (2007). Cytokinesis-block micronucleus cytome assay. *Nature Protocols*.

[54] Schmittgen T. D., Livak K. J. (2008). Analyzing real-time PCR data by the comparative C_T_ method. *Nature Protocols*.

