# Supplementary material for: Intracellular and Intercellular Signalling Mechanisms following DNA Damage Are Modulated By PINK1
Source: Oxid Med Cell Longev. 2018 Jun 27;2018:1391387. doi: 10.1155/2018/1391387 (PMC6079383; doi:10.1155/2018/1391387)
Supplement: Supplementary Materials — The Supplementary File contains six supplementary figures. Supplementary Figure 1: level of PINK1 downregulation in the cell lines used in the study and the effect of PINK1 loss of function on basal ATP level. Supplementary Figure 2: representative images of γ-H2AX/53BP1 staining. Supplementary Figure 3: representative images of micronuclear staining. Supplementary Figure 4: impact of BLM treatment on SH-SY5Y viability, caspase activation, oxidative species accumulation, and ATP. Supplementary Figure 5: compartmental stress signalling does not appear to be affected by BLM. Supplementary Figure 6: PINK1 loss of function impairs transmission of intercellular, bystander signalling following X-ray irradiation. [file 1391387.f1.pdf]

Intracellular and intercellular signalling mechanisms following DNA damage are modulated by PINK1.

MihaelaTemelie[1], Diana Iulia Savu[1,3], Nicoleta Moisoi[2,3]

1 Department of Life and Environmental Physics, Horia Hulubei National Institute of Physics and Nuclear Engineering, Reactorului 30, P.O. Box MG-6, Magurele 077125, Romania

2 Nicoleta Moisoi: Leicester School of Pharmacy, Faculty of Health Sciences, De Montfort University, The Gateway, Hawthorn Building 1.03, LE1 9BH, Leicester, UK,

3 Corresponding authors:
Nicoleta Moisoi: Leicester School of Pharmacy, Faculty of Health Sciences, De Montfort University, The Gateway, Hawthorn Building 1.03, LE1 9BH, Leicester, UK, Tel: +44 (0) 116 2078985,e.mail: nicoleta.moisoi@dmu.ac.uk
Diana Iulia Savu: Department of Life and Environmental Physics, Horia Hulubei National Institute of Physics and Nuclear Engineering, Reactorului 30, P.O. Box MG-6, Magurele 077125, Romania, tel: +40214046134; e.mail: dsavu@nipne.ro

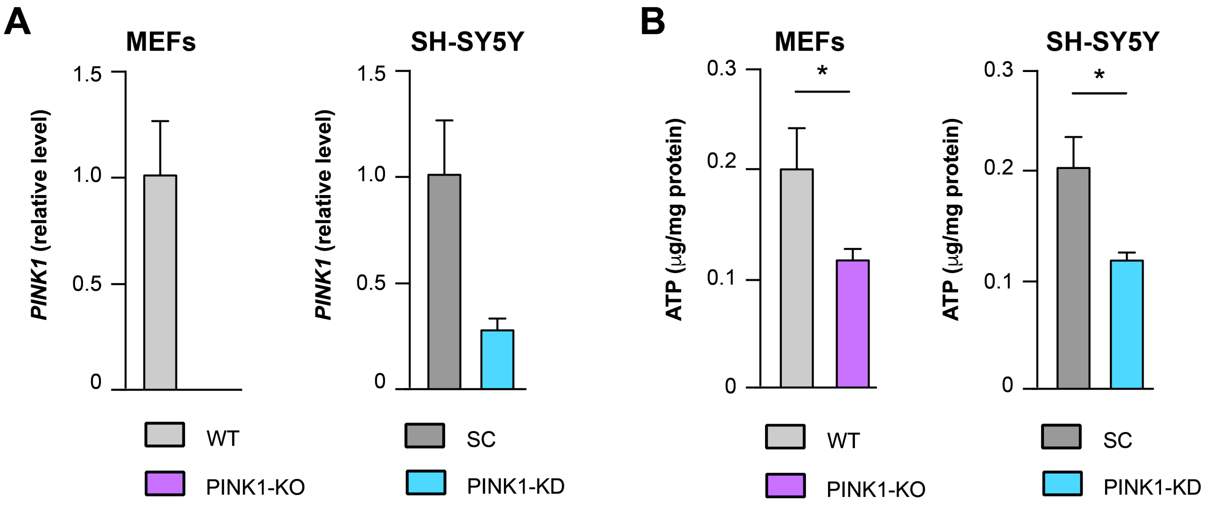

Supplementary Figure S1.

A. The genotype of the cell lines employed was verified by qRT PCR using PINK1 specific primers (mouse for MEFs and human for SH-SY5Y). The MEFs PINK1-KO did not present any signal confirming the deletion of the PINK1 gene. The downregulation in PINK1 mRNA levels achieved by shRNA in the human SH-SY5Y gene achieved 75%. Each data point represents the mean ± SEM of at least three independent experiments.

B. The basal ATP level as measure of mitochondrial function is diminished in the cells with PINK1 loss of function. Each data point represents the mean ± SEM of at least three independent experiments. Statistical analysis is performed with Student t-test; * $p<0.05$.

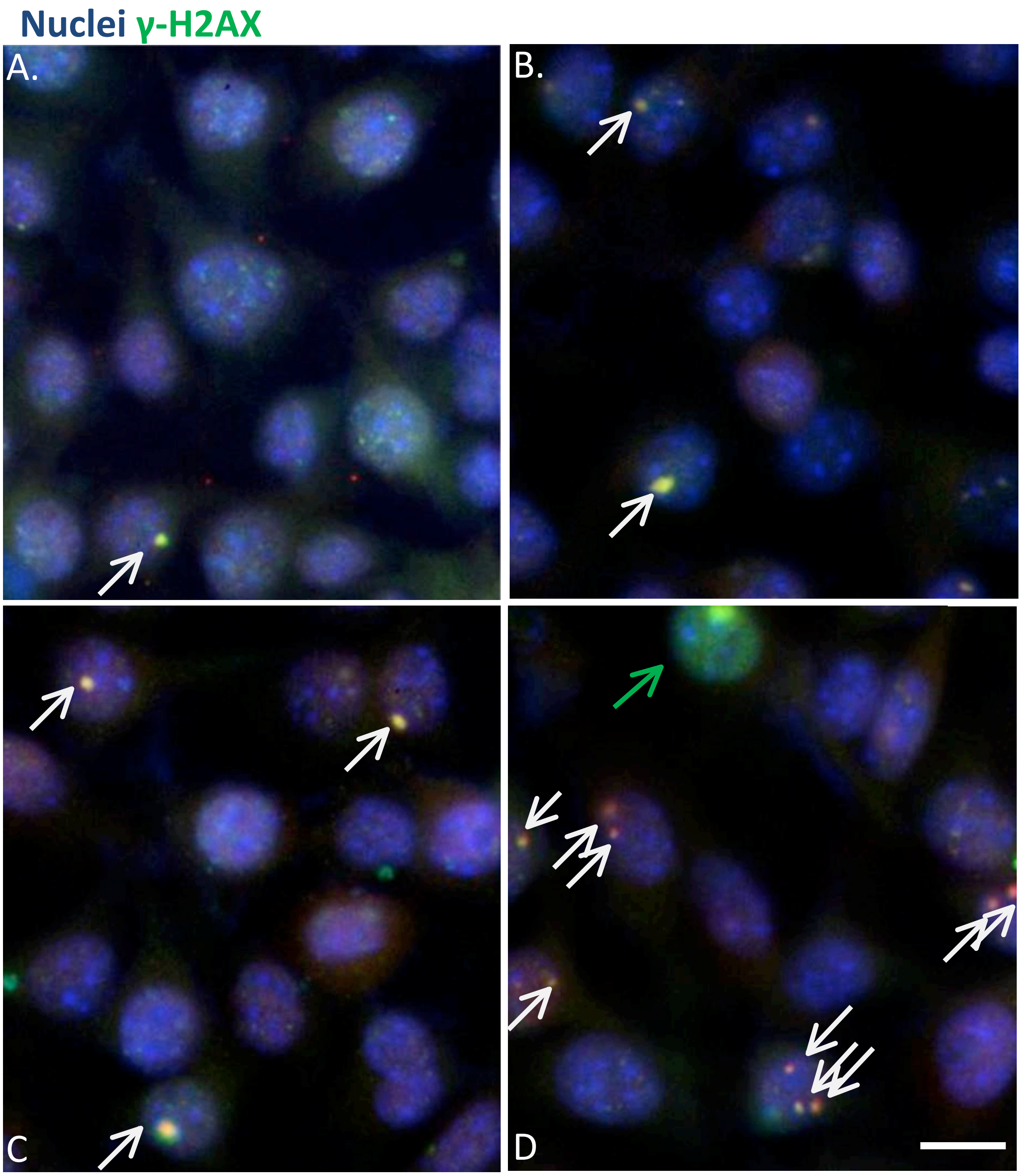

Supplementary Figure S2. Representative images of γ-H2AX/53BP1 staining. White arrows show γ-H2AX/53BP1 foci. The green arrow shows a cell fully saturated with γ-H2AX *Scalebar 10μm.* **A**. MEF WT - control; **B**. MEF PINK1 KO - control; **C**. MEF WT - BLM 40 μg/ml; **D**. MEF PINK1 KO - BLM 40 μg/ml;

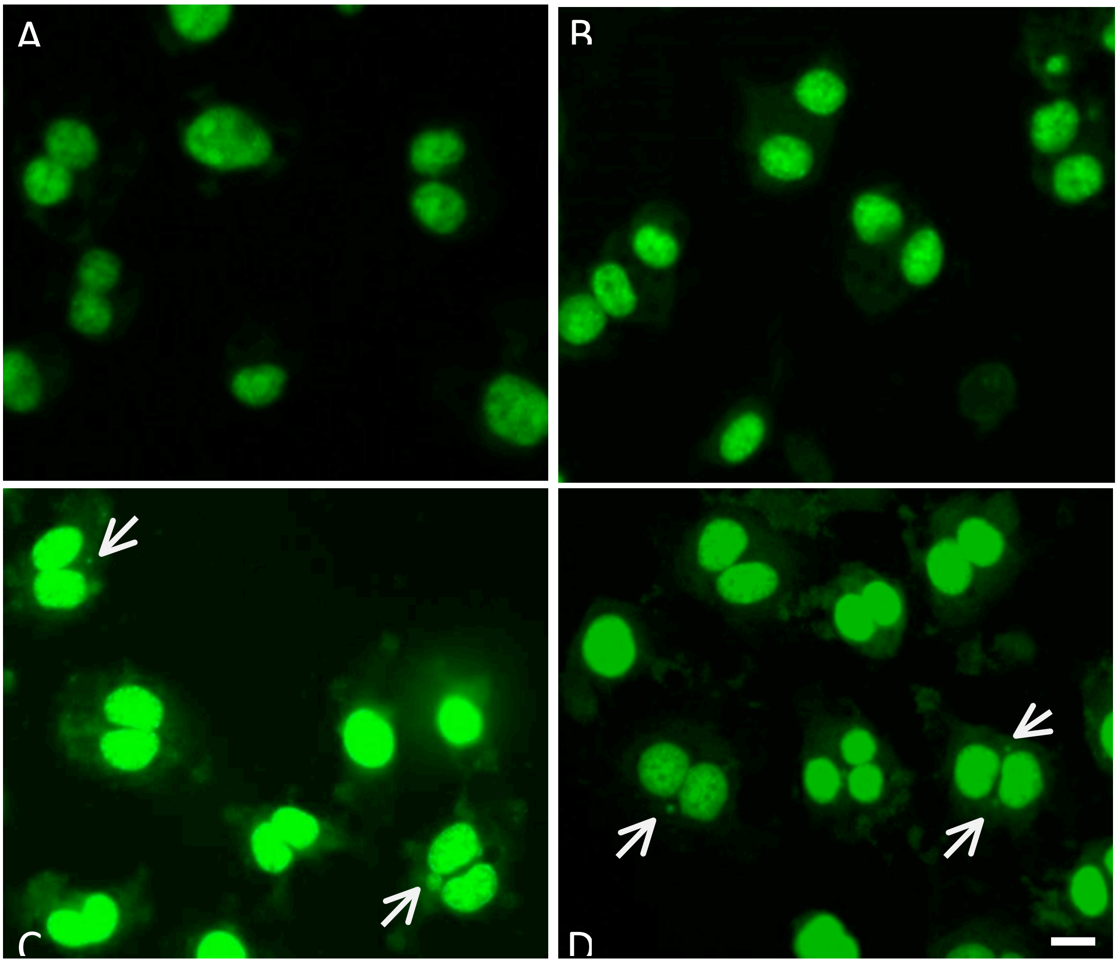

Supplementary Figure S3. Representative images of micronuclei staining. White arrows show micronuclei in binucleated cells. *Scalebar 10μm.* **A**. MEF WT - control; **B**. MEF PINK1 KO - control; **C**. MEF WT - BLM 40 μg/ml; **D**. MEF PINK1 KO - BLM 40 μg/ml;

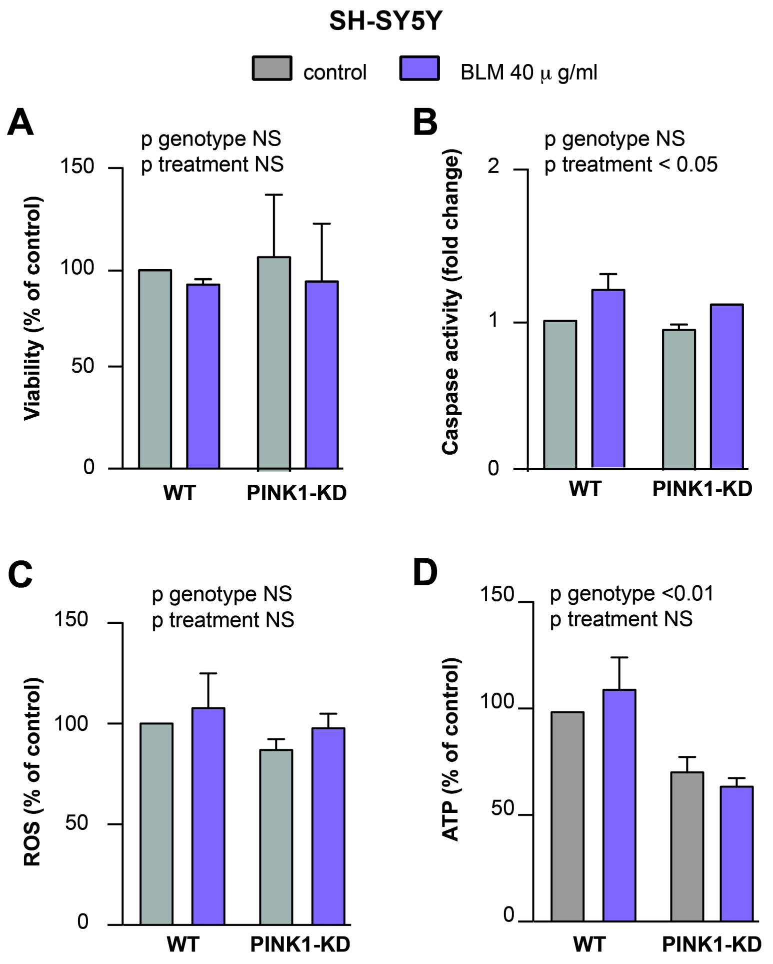

Supplementary Figure S4.

Treatment of SH-SY5Y with 40 µg/ml BLM has lower impact on the end-points analysed as compared to the MEFs cells. Viability assessed by the MTS test did not change significantly (**A**), while there is an increased activation of caspases as executors of programmed cell death (**B**). The ROS level does not seem to be influenced neither by genotype or treatment in these experimental conditions (**C**). The ATP level was not unmodified following the treatment with BLM (**D**). The data is reported as percentage of WT untreated control or fold change versus WT untreated control. Each data point represents the mean ± SEM of at least three independent experiments. Statistical analysis is performed with Two Way ANOVA with multiple comparisons and p-values are indicated in the figure.

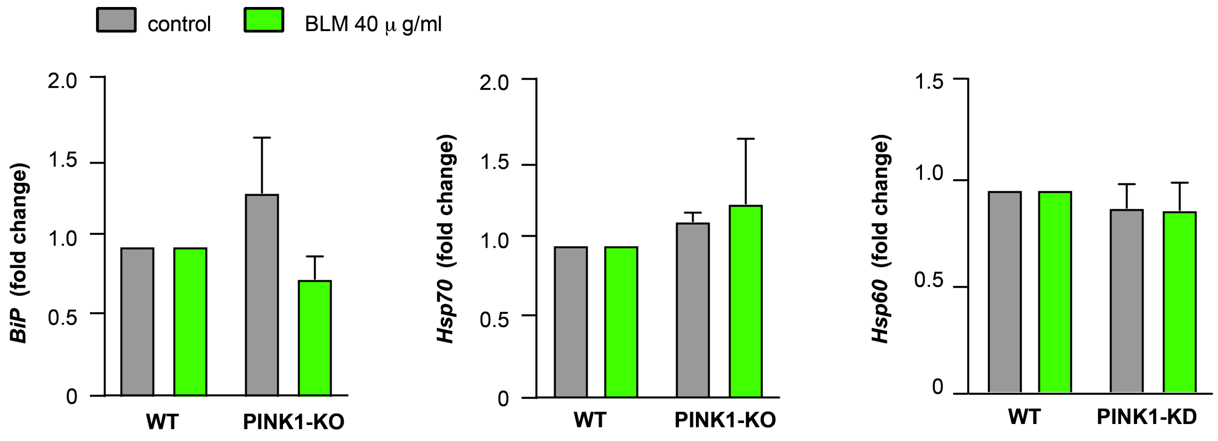

Supplementary Figure S5.

MEF cells from PINK1 KO and WT control have been treated with BLM at 40 μg/ml for one hour and the cells were harvested 24 hours later for analysis of transcriptional changes in cellular stress signalling factors. None of the stress signalling factors tested ER stress (BiP), cytoplasmic stress (Hsp70) or mitochondrial stress (Hsp60) did not present significant changes induced by BLM treatment. The data is reported as fold change versus untreated control for the corresponding genotype. Each data point represents the mean ± SEM of at least three independent experiments and it is presented as the change relative to control for each genotype.

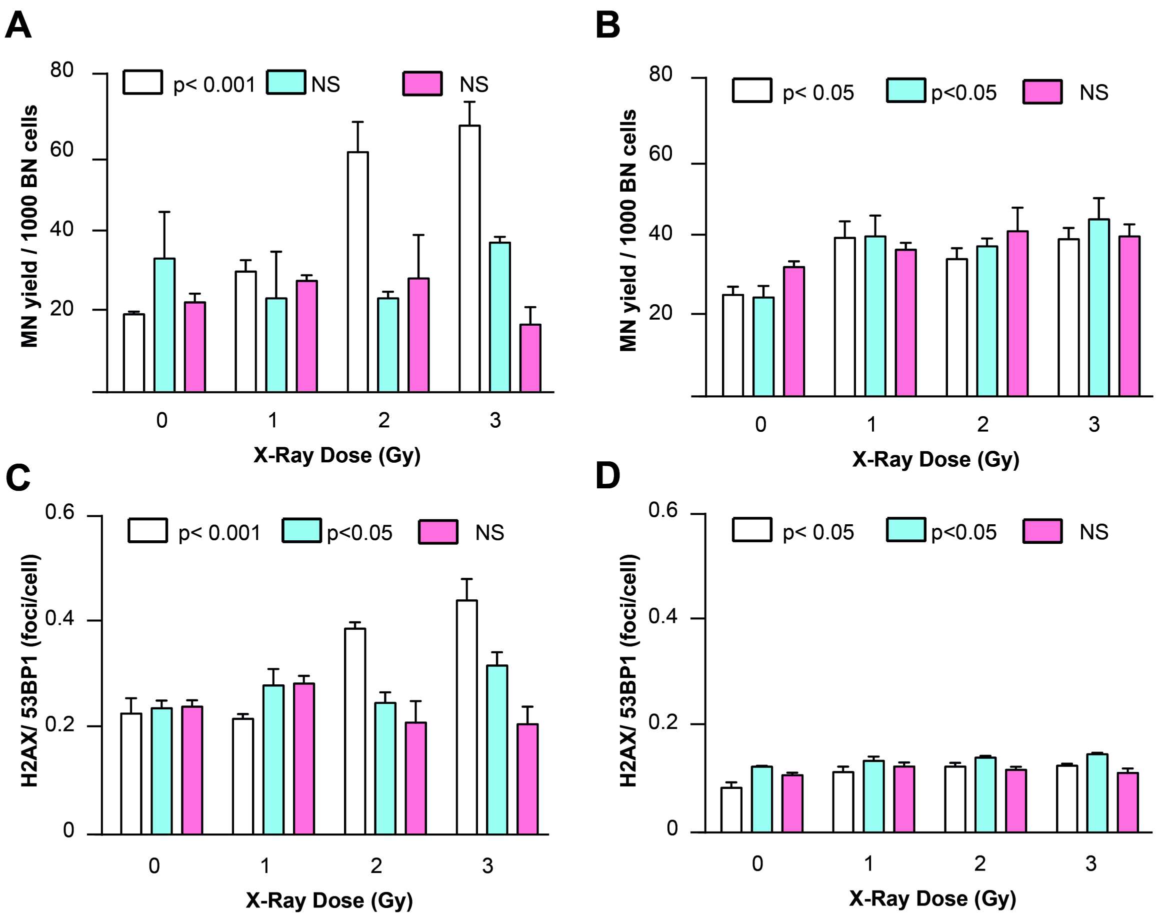

Supplementary Figure S6.

For the direct treatment the cells were exposed to X-ray then washed and incubated with fresh medium. This medium was conditioned for 24 hours with bystander factors by the directly treated cells and was used for the study of the bystander effect. The bystander cells are grown in medium transferred from X-ray treated donor cells collected at 24h post-treatment. The transfer of media was performed between MEFs WT and KO or SH-SY5Y SC and KD as follows. The medium was transferred either from WT/SC to WT/SC, WT/SC to PINK1 KO/KD or from PINK1 KO/KD to WT/SC cells. (**A, C**) In MEFs, the MN induction and the number of γH2AX/53BP1 foci increase with the irradiation dose only when the WT cells are grown 24h in medium transfer from WT cells. (**B, D**) For SH-SY5Y, the MN yield and γH2AX/53BP1 foci accumulated in both bystander SC and PINK1 KD cells grown in medium transfer from SC cells. Each data point represents the mean ± SEM of at least three independent experiments. P values from One Way ANOVA are indicated in the figure.
